# Clean room microbiome complexity impacts planetary protection bioburden

**DOI:** 10.1186/s40168-021-01159-x

**Published:** 2021-12-04

**Authors:** Ryan Hendrickson, Camilla Urbaniak, Jeremiah J. Minich, Heidi S. Aronson, Cameron Martino, Ramunas Stepanauskas, Rob Knight, Kasthuri Venkateswaran

**Affiliations:** 1 Biotechnology and Planetary Protection Group, Jet Propulsion Laboratory, California Institute of Technology, Pasadena, USA; 2 Marine Biology Research Division, Scripps Institute of Oceanography, University of California San Diego, La Jolla, CA USA; 3 Center for Microbiome Innovation, University of California San Diego, La Jolla, CA USA; 4 Bioinformatics and Systems Biology Program, University of California San Diego, La Jolla, CA USA; 5 Bigelow Laboratory for Ocean Sciences, East Boothbay, ME USA

## Abstract

**Background:**

The Spacecraft Assembly Facility (SAF) at the NASA’s Jet Propulsion Laboratory is the primary cleanroom facility used in the construction of some of the planetary protection (PP)-sensitive missions developed by NASA, including the Mars 2020 Perseverance Rover that launched in July 2020. SAF floor samples (*n*=98) were collected, over a 6-month period in 2016 prior to the construction of the Mars rover subsystems, to better understand the temporal and spatial distribution of bacterial populations (total, viable, cultivable, and spore) in this unique cleanroom.

**Results:**

Cleanroom samples were examined for total (living and dead) and viable (living only) microbial populations using molecular approaches and cultured isolates employing the traditional NASA standard spore assay (NSA), which predominantly isolated spores. The 130 NSA isolates were represented by 16 bacterial genera, of which 97% were identified as spore-formers via Sanger sequencing. The most spatially abundant isolate was *Bacillus subtilis*, and the most temporally abundant spore-former was *Virgibacillus panthothenticus*. The 16S rRNA gene-targeted amplicon sequencing detected 51 additional genera not found in the NSA method. The amplicon sequencing of the samples treated with propidium monoazide (PMA), which would differentiate between viable and dead organisms, revealed a total of 54 genera: 46 viable non-spore forming genera and 8 viable spore forming genera in these samples. The microbial diversity generated by the amplicon sequencing corresponded to ~86% non-spore-formers and ~14% spore-formers. The most common spatially distributed genera were *Sphinigobium*, *Geobacillus*, and *Bacillus* whereas temporally distributed common genera were *Acinetobacter*, *Geobacilllus*, and *Bacillus*. Single-cell genomics detected 6 genera in the sample analyzed, with the most prominent being *Acinetobacter.*

**Conclusion:**

This study clearly established that detecting spores via NSA does not provide a complete assessment for the cleanliness of spacecraft-associated environments since it failed to detect several PP-relevant genera that were only recovered via molecular methods. This highlights the importance of a methodological paradigm shift to appropriately monitor bioburden in cleanrooms for not only the aeronautical industry but also for pharmaceutical, medical industries, etc., and the need to employ molecular sequencing to complement traditional culture-based assays.

Video abstract

**Supplementary Information:**

The online version contains supplementary material available at 10.1186/s40168-021-01159-x.

## Introduction

Planetary protection (PP) is a scientific discipline aimed at preventing microbial contamination on spacecraft outbound to a celestial body (forward planetary protection) and on spacecraft hardware and samples returning to Earth (backward planetary protection) to avoid harmful contamination to a native environment or compromising scientific data. While sampling spacecraft surfaces for research purposes is challenging due to extremely low biomass and limited hardware availability, the cleanrooms in which spacecraft is assembled can act as a suitable surrogate for understanding microbial communities related to PP missions [1–6] and identifying microbes that could pose forward contamination risk. The Spacecraft Assembly Facility (SAF) at NASA’s Jet Propulsion Laboratory (JPL) is an ISO 8 (Class 100,000) certified cleanroom which has strict environmental controls such as temperature (20 ± 4°C), humidity (30 ± 5%), restricted human access, and stringent cleaning regimes [7, 8]. The SAF has been used for many years to assemble various interplanetary spacecraft, including the recently launched Mars 2020 Perseverance rover.

The microbial burden of past PP sensitive spacecraft hardware has been determined through spore detection via the NASA standard assay (NSA), which involves collecting samples using swabs or wipes from surfaces, heat shocking at 80°C for 15 min, plating on Tryptic Soy Agar (TSA), and incubating the plates for up to 72 h at 32°C [9–19]. Although the NSA can provide an estimate of biological cleanliness on a spacecraft, the various selective conditions in the NSA method select for a narrow range of aerobic bacteria. Thus, the presence of other spore-formers (i.e., non-NSA spore-formers) and the remaining viable microbial population that are relevant to PP are underestimated [9, 17, 20]. These underestimated microbial populations can be determined via culture-independent molecular methods [21] or approximated using a conversion ratio between the NSA spore-formers and the viable microbial population. This ratio, as outlined by the Space Studies Board (SSB), is 50,000 viable cells for every 1 NSA spore [22]. A recent publication of the SAF cleanroom environment revealed that the ratio of NSA spores to viable microbial cells was in the range of 150 to 12,000 depending on the methodology used [23]. Although this earlier SAF report quantitatively measured the microbial burden in SAF, additional research was required to gain a more thorough understanding of the microbial community composition of the SAF.

In 2011, the NSA and DNA microarray methods were compared for their ability to assess relative bacterial abundance and diversity on spacecraft surfaces [6]. Cultivable spore-forming bacterial counts derived from the NSA were extremely low for spacecraft surfaces. However, the PhyloChip generation 3 (G3) DNA microarray resolved the genetic signatures of a highly diverse suite of microorganisms in the very same sample set [6]. Samples completely devoid of cultivable spores were shown to harbor the DNA of more than 100 distinct microbial phylotypes. Furthermore, samples with higher numbers of cultivable spores did not necessarily give rise to a greater microbial diversity upon analysis with the DNA microarray [6]. The findings of this comparative study clearly demonstrated that there is not a statistically significant correlation between the cultivable spore counts obtained from a sample and the degree of bacterial diversity present [6]. Since 2011, several technological advancements emerged including extracting nucleic acids [24] and analyzing the target molecules using the next-generation sequencing (NGS) methods [1, 25]. In the present study, a direct comparison of 16S rRNA gene-targeted amplicon sequencing of the NSA isolates (spores) using Sanger sequencing with the next-generation sequencing of the 16S rRNA gene (all bacteria) was attempted to understand any correlation between incidence of bacterial spore-formers and total bacterial diversity. Since it has been established that spacecraft and associated surfaces have a negligible percentage of archaea (<1%) [26] and fungi (~2 to 4%) [27], only bacterial diversity was measured during this study.

Thus, the objective of this study was to perform an in-depth analysis of the SAF facility microbial community composition over the course of 6 months, using 16S rRNA gene-targeted amplicon sequencing to understand spatial and temporal relationship of the spore-forming members and bacterial diversity. Furthermore, the amplicon sequencing datasets were analyzed to understand the significance of the members of spore-formers not detected by the NSA method as well as diversity of non-spore forming bacteria. In addition, the fluorescence-activated cell sorting (FACS) and subsequent single-cell genomics (SCG) were tested for the feasibility of cultivation-independent quantification, taxonomic identification, and genome analyses of viable microorganisms in some of the low-biomass cleanroom samples. Despite several previous studies that have investigated the microbial diversity in NASA cleanrooms during various missions, this is the first-time a large number of samples were collected throughout a 6-month time period to comprehend the core bacteriome of the assembly facility, in addition to assessing whether the cultivation-dependent NSA spore assay is a good proxy for predicting the total viable bacterial population.

## Methods

### Sample collection and processing

**Fig. 1:**
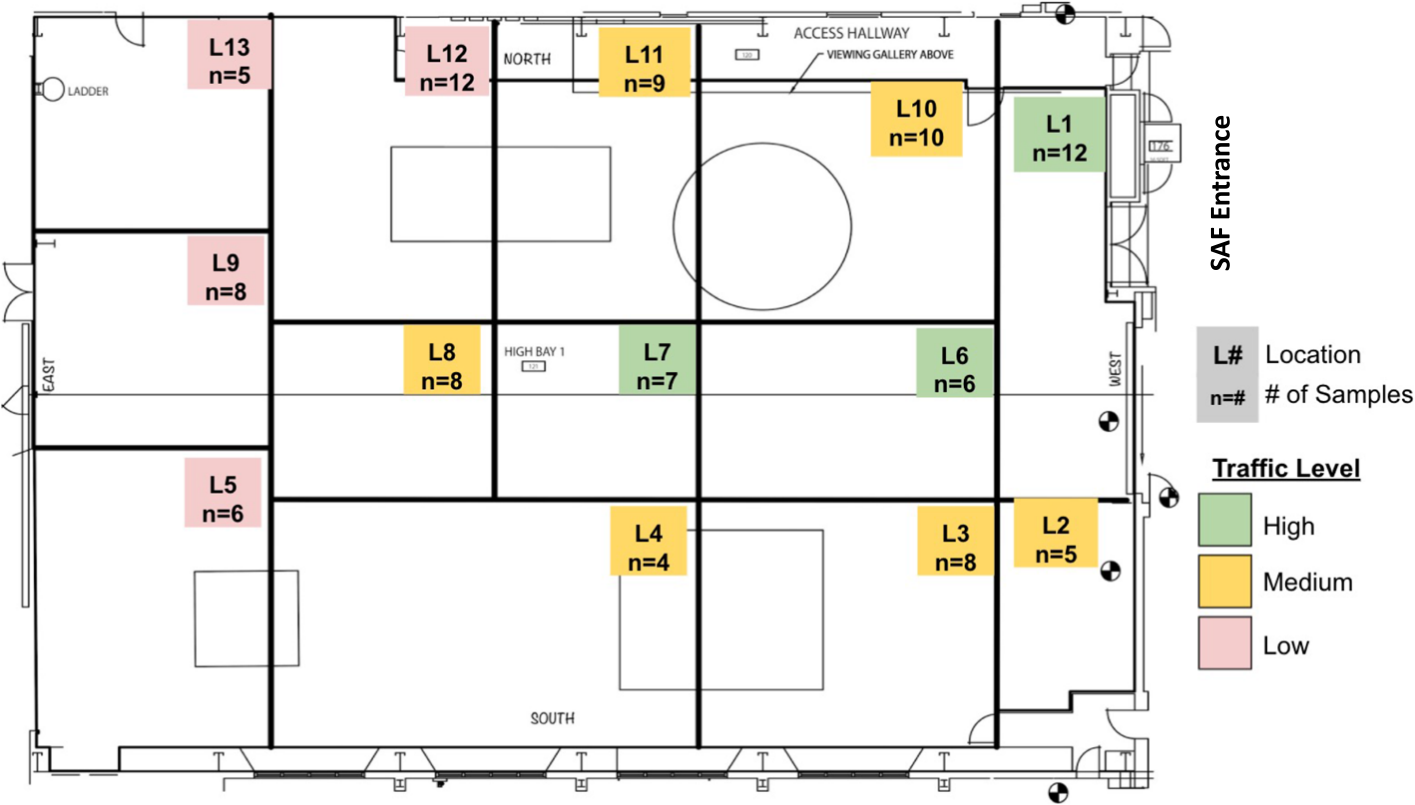
Schematic of the dates and locations sampled in the Spacecraft Assembly Facility. A total of 98 samples were collected over a 6-month period from the SAF. The graph is compartmentalized into artificial section based on sample grouping and foot traffic. Each section describes the location number and number of samples collected throughout the study in a gray box. In total, there are 11 sampling dates and 13 sampling locations. The sample collection was carried out between March 2016 and August 2016, and 98 floor samples were collected during 11 sampling time periods in the JPL SAF. Total surface area of the SAF cleanroom is 921.1 m^2^ with controlled conditions such as temperature (20 ± 4°C), humidity (30 ± 5%), stringent gowning requirements, and weekly cleaning [28]. Although SAF is capable of becoming an ISO-7 (10k) cleanroom, at the time of sampling SAF was certified as an ISO-8 (100k) cleanroom. A maximum measurement of 8287, 0.5 μm particles/ft^3^ and 159, 5.0 μm particles/ft^3^ were seen during the 6 months of the study. High traffic area: L1, L6, and L10; low traffic area: L5, L9, and L13. The rest of the locations had moderate traffic due to hardware assembly

Over a period of 6 months, between March 2016 and August 2016, 98 floor samples were collected during 11 sampling sessions in the SAF. The specific locations for each sampling event are given in Fig. 1. Floor samples of 1 m^2^ were collected using sterile, pre-moistened 9” × 9” polyester wipes (Texwipe; TX1009, NC, USA) as previously described [29]. The sampling was performed at the same location as much as possible within 2-to-3-m area over the period of 6 months. Locations were chosen at each sampling event to capture representative areas from high, medium, and low trafficked areas, as well as availability of floor space not blocked by hardware or other equipment.

Immediately after sample collection, wipe samples were deposited into sterile 500-mL glass bottles and transferred to the lab for further processing [29, 30]. Once in the lab, 200-mL of sterile phosphate-buffered saline (PBS; pH 7.4; Sigma Aldrich, MO, USA) solution was added and vigorously shaken for 30 s to thoroughly mix the solution and release any collected particulates and associated microorganisms. The 200-mL sample was then concentrated to approximately 5 mL using an InnovaPrep concentrating pipette with 0.45-μm hollow fiber polysulfone (HFPS) concentrating pipette tips (InnovaPrep Drexel, MO, USA). The exact amount of concentrated sample was weighed on a tared scale and appropriately recorded. The concentrated samples were then used for several culture-dependent and culture-independent assays. The microbial burden of the samples using the cultivation assays (spores and cultivable aerobic bacteria) and viability assays (adenosine triphosphate (ATP), Propidum Monoazide quantitative polymerase chain reaction (PMA-qPCR)) were performed as previously described [23].

### 16S rRNA amplicon Sanger sequencing of spore-forming isolates

A modified NSA procedure was performed on each sample to measure the cultivable, heat shock-resistant spore population. Procedures varied slightly from the standard NSA procedure [29]. An aliquot of 425 μl from the 5-mL concentrated sample was heat shocked at 80°C ± 2°C for 15 min. After cooling to room temperature, 100-μl aliquots were deposited into four individual sterile petri dishes for quadruplicate measurements. Molten, sterile TSA (Thermo Fisher Scientific, Chino, CA) was then added using an aseptic standard plate pouring technique. Once solidified, plates were incubated at 32°C and colony forming units (CFUs) were counted after 24 h, 48 h, 72 h, and 7 days of incubation time. A total of 130 individual colonies from the NSA were successfully stored in semi-solid TSA as stab cultures at room temperature for further analyses.

Heat-shocked isolates were grown from stab cultures on TSA overnight at 30°C, and DNA was extracted using the Mo Bio UltraClean Microbial DNA Isolation Kit (Mo Bio Laboratories, Carlsbad, CA). The extracted DNA was used to amplify the 1.5 kb 16S rRNA gene using the following primers: 27F (5’-AGA GTT TGA TCC TGG CTC AG-3’) and 1492R (5’-GGT TAC CTT GTT ACG ACT T-3’). PCR conditions and Sanger sequencing parameters were followed as established before [31, 32]. Sequence data were processed and trimmed using DNASTAR SeqMan Pro and sequences were identified using the SILVA LTP type strain SSU database (version 132). Sequences were aligned using MUSCLE [33], and a maximum likelihood phylogenetic tree was reconstructed using FastTree [34]. Novel species were determined using a 98.7% sequence similarity cutoff [35]. 16S rRNA gene sequences were deposited to GenBank under the accession numbers MW130960–MW131089.

### PMA treatment

To distinguish between viable and non-viable cells, samples were treated with PMA, a DNA-intercalating dye [36]. Of the concentrated 5mL sample, a single 1.5-mL aliquot was treated with 18.75 μL of 2 mM PMA (Biotium, Inc., Hayward, CA, USA). This aliquot (PMA-treated) and another 1.5 mL aliquot (non-PMA-treated) were vortexed and incubated in the dark for 5 min at room temperature. Both aliquots were then exposed to PhAST Blue-Photo activation system (GenIUL, S.L., Terrassa, Spain) for 15 min [8, 37–39]. DNA was then extracted from samples using the Maxwell DNA extraction system (Promega, Madison, WI, USA). DNA suspensions for both conditions were stored in molecular grade water (50 μl each) at –20°C for further analysis [23, 40].

### 16S rRNA amplicon Illumina sequencing analysis

A total of 236 samples were processed for 16S rRNA sequencing. Samples were distributed across four sample types including floor wipes (*n*=196; 98 PMA-treated, 98 PMA naive), field controls of wipes exposed to environment (in situ air) (*n*=14; 7 PMA-treated, 7 PMA naive), negative controls (wipes only) (*n*=14; 7 PMA-treated, 7 PMA naive), and Maxwell controls (DNA extraction reagents) (*n*=8; 8 PMA naive).

The V4 region of the 16S rRNA gene was amplified using the 515f/806r primers and prepared for sequencing using the protocol described in the Earth Microbiome Project [41]. Libraries of 16S rRNA were prepared using equal volumes of DNA and were pooled at equal volumes post PCR to enable comparison of read counts across samples as read counts correlated to input biomass without distorting composition [1]. Final DNA concentration was ~100 ng post PCR and ~1 ng before PCR. Sequencing was performed at the UC San Diego Institute for Genomic Medicine facility using a HiSeq2500 Rapid run. Since the amount of microbial biomass (cells) [1] and subsequent DNA in the original sample correlates to the read counts post sequencing [42], it is absolutely critical to ensure equal volumetric pooling of libraries prior to sequencing, particularly when working with low biomass samples.

Raw reads were demultiplexed and quality filtered using QIIME2 v2019.10 with default parameters [43]**.** Quality-filtered reads were clustered into sub-Operational Taxonomic Units (sOTUs) using deblur [44]. A phylogenetic tree was constructed through SEPP insertion [45] with the Greengenes v13_8 as a reference backbone [46]**.** To identify and remove background contaminant sOTUs, sequences which were present in 100% of controls and were observed in higher absolute abundance in no-template controls than in samples were removed from the dataset. A stringent removal protocol based on presence in no-template controls alone was not used because sOTUs which are found in negatives are often a result a well-to-well contamination from nearby samples [46].

The resulting table was rarefied to 5000 counts per sample. Of the 198 primary samples (99 processed through PMA- (naïve) or PMA + (treated), 153 were successful (92 PMA-, 61 PMA treated) making up 12,825 sOTUs. Of the 38 controls, 31 had at least 5000 reads making up 983 unique sOTUs. Of those 983 sOTUs found in negative controls, 894 were found in less than 10% of the samples. Seven sOTUs, however, were present in all controls and thus deemed as putative reagent or processing contaminants.

Within sample type comparisons of read counts or alpha diversity was compared using Kruskal-Wallis test followed by Benjamini Hochberg multiple comparisons post hoc testing [47, 48]. Beta diversity comparisons were performed using unweighted and weighted UniFrac [49, 50]. Dimensionality reduction on the UniFrac distances was performed through Principal Coordinates Analysis (PCoA). Robust Aitchison Principal component analysis (RPCA) was performed on non-rarefied data and used to produce an Aitchison distance matrix [51]**.** Multivariate statistical testing of drivers of distance metrics were performed using Permutational multivariate analysis of variance (PERMANOVA) [52].

Additionally, 184 out of 236 samples tested passed through the quality control measures and were used for further analysis. This included floor wipes (*n*=153; 61 PMA-treated, 92 PMA naive), field controls of wipes exposed to environment (*n*=11; 4 PMA-treated, 7 PMA naive), negative controls (wipes only) (*n*=12; 6 PMA-treated, 6 PMA naive), and Maxwell controls (DNA extraction negatives) (*n*=8; 8 PMA naive). Heatmap visualizations and differential abundance comparisons between PMA- and PMA+ treated samples were performed using Calour [53] and specifically with the rank-based DA testing which utilizes a discrete false discovery rate [54].

### Compositional tensor factorization and log-ratios

In the subset of PMA-treated data, compositional tensor factorization (CTF) (v. 0.0.5) was used to account for the repeated measure structure of the longitudinal measurement and the compositional nature of microbiome data [55]. Based on feature loadings produced from the unsupervised RPCA [51] and CTF [55] analyses (e.g., biplots), log-ratio of taxonomic groups determined by the lowest common ancestor were produced through Qurro [56]**.** The significance of log-ratios between categorical groupings was determined through a two-sided *T* test with Bonferroni multiple-comparison correction when appropriate. For comparisons of log-ratio to continuous measurements (e.g., radius), a Pearson linear correlation test was used. All statistical testing was performed using Scipy (v. 0.14.0) [57].

### Effect size analysis

In order to calculate the relative effect size of all recorded metadata within the RCPA and UniFrac ordinations, a stepwise redundancy analysis (RDA) was performed. The stepwise analysis was performed on the first three principal components of each ordination through the ordistep function in vegan v2.4-2 [58]**.** The ordistep function was run following the established procedure [59].

### Sporulation prediction

In order to predict the possible sporulation of observed sOTUs, we performed an ancestral state reconstruction using the BacDive bacterial diversity metadatabase as the training set [60]**.** First, microbial strain entries with both spore formation and full 16S marker gene sequence information available from the BacDive database were downloaded. The full 16S sequence for each strain was downloaded with Entrez by the (National Center for Biotechnology Information) NCBI ID. Each sequence was matched exactly (i.e., 100% identity) to the Greengenes phylogeny (v. 13_8) [61] which resulted in 9004 matches between the BacDive data and Greengenes phylogenetic tree. Next, sporulation ability prediction was performed through castor ancestral state reconstruction (v. 1.5.0) using empirical state probabilities across tips [62]**.** To evaluate this prediction, leave one out cross-validation was performed on each entry with sporulation information and precision-recall was evaluated, resulting in an average precision-recall of 0.82. Finally, the observed sOTUs from this study were predicted for the ability to produce spores using a SEPP insertion tree [45] with the sporulation-labeled Greengenes phylogenetic tree as the backbone for insertion.

### Source tracking

In order to determine the source environments contributing to variation in microbial signatures within the SAF facility, microbial source tracking was used in combination with the American Gut Project (AGP) and Earth Microbiome Projects (EMP) samples as sources [41, 63]. First, the AGP and EMP datasets and accompanying metadata used for source environments were downloaded through redbiom (v. 0.3.5) [64] with the context set to Deblur [44] generated data from 16S V4 variable region Illumina sequencing data trimmed to 150 base pairs. This source data was then merged with the SAF dataset representing the tracking sinks. A feature frequency filter was applied such that all features appear with at least 0.1% prevalence across the whole dataset. This resulted in a table of 10,095 samples by 55,640 sOTUs representing ten total Empo-3 source environments. This dataset was then used for microbial source tracking with SourceTracker2 (v. 2.0.1-dev) [65] with default parameters.

### Single-cell genomics

After the initial InnovaPrep concentration of the sample (*n*=25), an aliquot was amended with 5% glycerol and 1 mM ethylene diamine tetraacetic acid (EDTA; final concentrations), stored at −80°C, and shipped on dry ice to the Bigelow Laboratory Single Cell Genomics Center (Maine, USA) for further processing. Once processing began, samples were diluted threefold with filtered (0.2-μm pore size) 1× PBS and stained with RedoxSensor Green (RSG; Thermo Fisher Scientific) to identify viable cells. Individual particles with reductase activity were identified by their high green fluorescence versus red fluorescence and sorted using an inFlux Mariner (BD) sorter into 384-well plates, containing 0.6 μl of Tris EDTA (TE) buffer per well, as previously described [66]. Each plate contained 317 single-cell, 64 no-cell (negative control), and three 10-cell (positive control) wells. Diameters of the sorted cells were determined using the fluorescence-activated cell sorting (FACS) light forward scatter signal, which was calibrated against cells of microscopy-characterized laboratory cultures [66]. Single-cell DNA amplification using whole genome amplification (WGA-X) and PCR-based sequencing of bacterial 16S rRNA genes were done on three microplates with cells from sample 2016-07-12:1, following previously described procedures [66]. Genomic sequencing of single amplified genomes (SAGs) from one of these plates was performed using previously described procedures [66]. Cell sorting and WGA-X were performed in a cleanroom environment. This workflow was previously evaluated for assembly errors using three bacterial benchmark cultures with diverse genome complexity and GC content (%), indicating no non-target and undefined bases in the assemblies and average frequencies of mis-assemblies, indels, and mismatches per 100 kbp being 0.9, 1.8, and 4.7 [66].

## Results

A schematic diagram of the SAF, where 98 samples from 13 different locations spanning 11 sampling sessions, is shown in Fig. 1. Only floor samples were collected because no flight hardware was available for this sampling campaign.

### Spore-forming bacterial composition

**Fig. 2:**
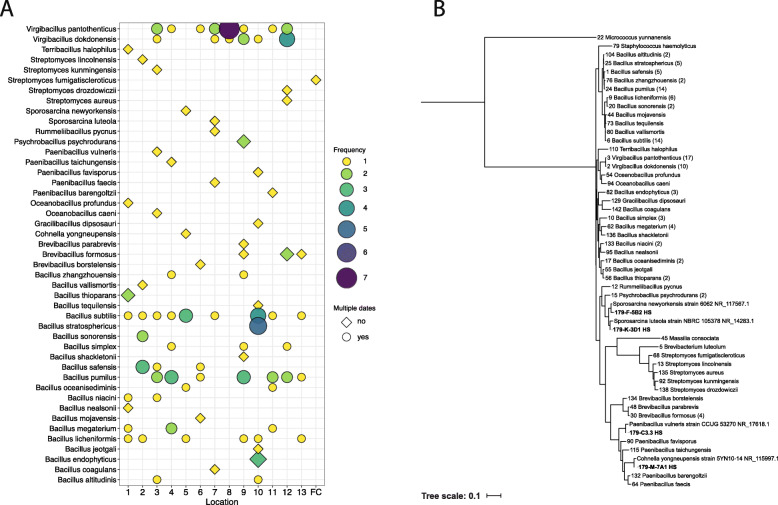
Sanger sequencing isolates from the NASA standard assay isolates. **A** Relative abundance of taxa at each location sampled in SAF. Dot size indicates the number of isolates recovered at a given location, while shape indicates whether an isolate was recovered on one sampling date (diamond) or multiple sampling dates (circle). The color of the dot indicates the number of isolates recovered at each location. **C** Phylogenetic tree of 16S rRNA genes from Spacecraft Assembly Cleanroom isolates. Numbers in parentheses indicate the number of isolates recovered for each species. Four novel (<98.7% sequence similarity) isolates were recovered (SAF Isolates 59, 66, 97, and 147) and are listed with their closest NCBI hit. The tree is based on maximum likelihood analysis and was constructed using FastTree

As reported earlier, the aerobic bacterial spore burden was 36 spores per m^2^ which is ~20% of the total heterotrophic cultivable bacterial counts [23]. Sanger sequencing of the full-length 16S rRNA gene on the NSA isolates from the 98 samples resulted in 130 isolates, belonging to 16 genera and 49 species (Fig. 2). 97% of these isolates (126/130) belonged to species of *Bacillus*, *Brevibacillus*, *Cohnella*, *Gracilibacillus*, *Oceanobacillus*, *Paenibacillus*, *Psychrobacillus*, *Rummeliibacillus*, *Sporosarcina*, *Streptomyces, Terribacillus*, and *Virgibacillus*, which are commonly considered spore-formers. *Bacillus* made up the majority of this population with 73 isolates belonging to 21 species. The remaining ~3% of the isolates cultured from the NSA (4/130) were non-spore forming bacteria consisting of *Brevibacterium luteolum*, *Massilia consociata*, *Micrococcus yunnanensis*, and *Staphylococcus haemolyticus*. When these isolates were re-tested for their tolerance to heat, they again showed survival upon plating after heat shock at 80°C for 15 min.

When analyzed spatially, 36 of the 49 identified species were not ubiquitous and isolated only once in a given SAF location. The most spatially abundant isolate was *Bacillus subtilis,* which was found in 9 out of the 13 sampled locations in SAF (Fig. 2a). When the isolates were analyzed temporally across the 11 sampling sessions, the majority of the species (30/49, ~61%) were isolated only once throughout the 6-month sampling period in a given sampling session. The most temporally abundant species were *Virgibacillus panthothenticus* (17 isolates) and *B. subtilis* (14 isolates), which were isolated from SAF floors on 7 individual sampling sessions (Fig. 2a) followed by *Bacillus pumilus* (14 isolates) that was found on 6 sampling sessions. The phylogenetic relationship amongst the cultured isolates is shown in Fig. 2a, with four isolates representing potentially novel species as they had ≤98.7% sequences similarity to the 16S rRNA sequence of any validly described species. Of the 16 genera identified, 3 were also identified in the 16S rRNA gene Illumina amplicon sequencing analysis with greater than 100 reads across all samples. Among the 3 genera identified with both methodologies one genus was a spore-former (*Bacillus)*, while the other two (*Massillia* and *Staphylococcus*) were non-spore-forming but heat tolerant.

### Molecular microbial composition of the SAF

**Fig. 3:**
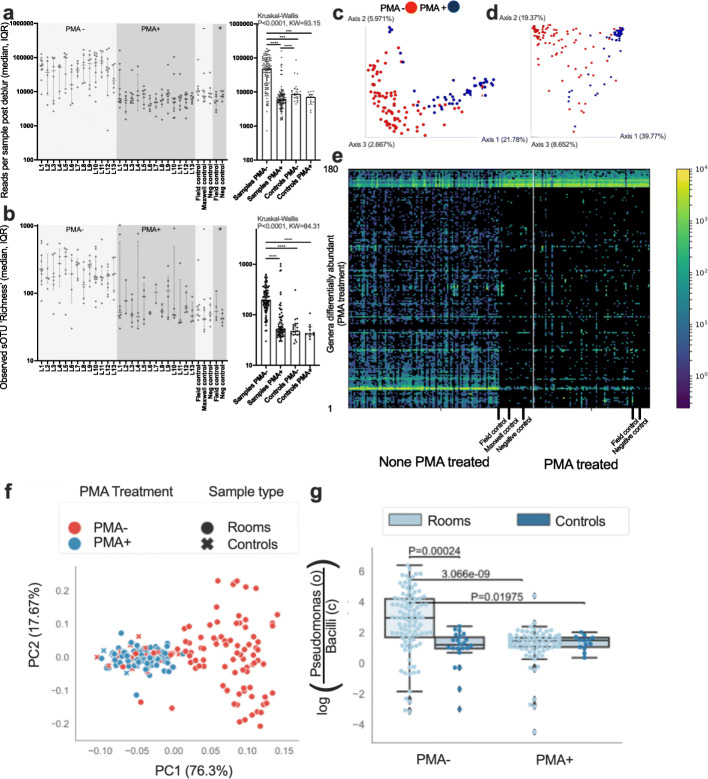
16S rRNA sequencing results (**A**–**B**). Distribution of **A** reads and **B** richness (alpha diversity) of 16S rRNA measured across the 13 SAF locations grouped by PMA treatment. Microbial composition of live vs dead cell communities as determined using PMA treatment (**C**–**E**). Microbial community beta diversity driven strongly by PMA treatment for **C** unweighted UniFrac and **D** weighted UniFrac (PMA- “red” and PMA+ treated “blue” samples). **E** Heatmap representation of the features (180 out of 1250 total genera) associated with PMA treatment as calculated using the differential abundance measure in Calour with controls labeled for reference. Comparison of microbial community composition between PMA live vs. dead treatment (**F**–**G**). Robust Aitchison PCA (RPCA) compare between PMA treated (blue) and untreated (red) SAF rooms (circles) and controls (ex) (**F**). Log-ratio of lowest common ancestor aggregated Pseudomonadales (order) and Bacilli (class) compared by PMA treatment between rooms (light blue) and controls (dark blue) (**G**). *P* values obtained from pairwise *t* tests with Bonferroni multiple comparisons correction

Per sample read counts differed across sample types (Kruskal-Wallis *P*<0.0001, KW=93.15) where PMA untreated samples had higher counts than both PMA-treated samples (*P*<0.0001) and controls regardless of treatment [PMA- (*P*<0.001) and PMA+ controls (*P*<0.001)] (Fig. 3a). Microbial richness also differed across sample types (Kruskal-Wallis *P*<0.0001, KW=84.31) with PMA untreated samples having higher richness than PMA-treated samples, and controls (*P*<0.0001) (Fig. 3b). Richness did not differ between PMA-treated samples or controls. The UniFrac unweighted (Fig. 3c) and weighted (Fig. 3d) analyses exhibited a differential beta diversity by PMA treatment. The heatmap representation of the features (180 out of 1250 total genera) associated with PMA treatment as calculated using the rank-based differential abundance measure in Calour, an interactive, microbe-centric analytical tool [53], is depicted in Fig. 3e, and it is evident that PMA treatment removed most of the dead microorganisms. When sequences were detected and compared from both PMA and non-PMA samples of a given sampling date and location, an average of ~33% of the OTU richness was determined to be from live cells or present due to background contamination in processing.

Changes in 16S rRNA gene amplicon sequencing composition were assessed through PCoA on unweighted and weighted UniFrac in addition to PCA on Aitchison distances produced from RPCA. PMA treatment and the collection timepoint led to significant differences in microbiota (Fig. 3f) and contributed to the majority of the total explained effect size in ordinations (Table S1). These shifts in beta-diversity by PMA treatment are partially attributed to a decrease in the log-ratio of *Pseudomonadales* (order) relative to *Bacilli* (class) (Fig. 3g). The log-ratio of *Pseudomonadales* (order) relative to *Bacilli* (class) was used to understand the microbial diversity shift because reads associated with *Pseudomonadales* were not increased in the sampled environment, while *Bacilli* reads were enriched by the entrance versus the interior of the facility.

To account for the repeated measure structure of the data (i.e., time), CTF was used to explore microbial community changes between SAF locations. CTF but not RPCA, weighted, or unweighted -UniFrac beta-diversity distances revealed significant differences between SAF room location (Table S2). The differences in rooms were explained partially by the log-ratio of *Rhizobiales* (Proteobacteria) to *Bacillales* (Firmicutes) (Fig. S1a; Fig. S1b). As the radius from the facility entrance increased, a decrease in the log-ratio of *Rhizobiales* (Proteobacteria) to *Bacillales* (Firmicutes) was also observed. We further hypothesized, given the known sporulation ability of *Bacillales* that the resilience as spores could possibly be driving the observed variation between SAF room location. Both *Rhizobiales* and *Bacillales* are often found in the environment and so could easily be brought in from external sources. But *Rhizobiales* are non-spore-forming and are likely non-viable far from the entrance, thus, the change in the ratio between the two was noticed and computed.

**Fig. 4:**
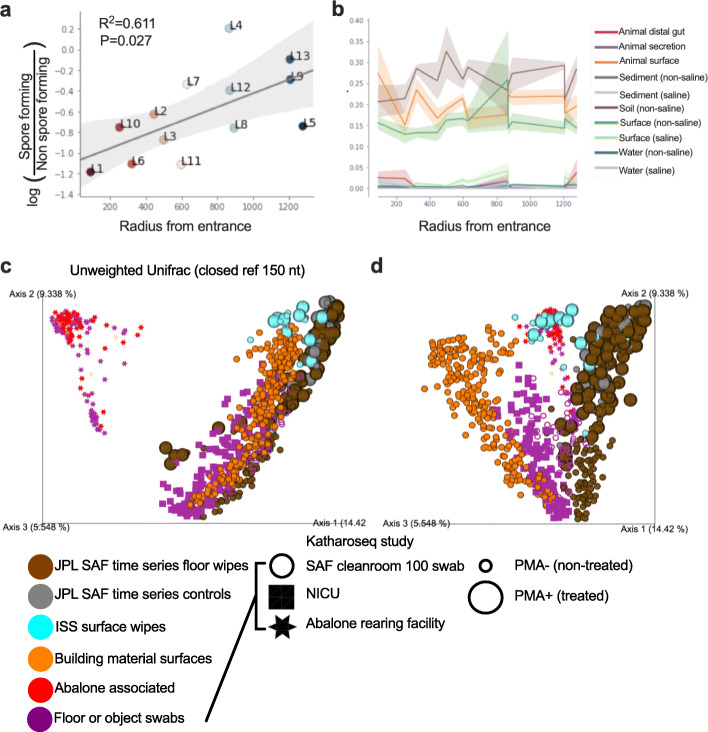
Microbial differentiation across SAF locations differs by radial distance from facility entrance (**A**–**B**). (*x*-axes) Linear regression plot of log-ratio of ancestral state predicted sporulating and non-sporulating bacteria (*y*-axis) by radial distance from entrance (**A**). Proportion of contribution from AGP and EMP empo-3 sources (*y*-axis) across radius from entrance (**B**). Pearson correlation used for linear comparison and error bars represent the standard error of the mean. **C** Unweighted (**C**) and weighted (**D**) microbiome meta-analysis of JPL SAF time series compared to other built environments (JPL SAF 100 swab study, International Space Station, building materials study, abalone rearing facility, NICU hospital study).

Using ancestral state reconstruction with the Deutsche Sammlung von Mikroorganismen und Zellkulturen (DSMZ) BacDive database, sporulation ability was predicted through the SEPP insertion tree (Average Precision=0.83; Fig. S2). The location mean log-ratio of predicted non-spore forming and spore forming microbes correlated with the radial distance from the entrance of the SAF facility by Pearson correlation (*r*=0.61, *P*=0.027; Fig. 4a) showing that the number of spore-formers compared to non-spore-formers decreases the further away from the entrance. Microbial source tracking of each room over time using the AGP and EMP datasets of sources revealed possible sources of SAF contaminant bacteria. As the radius from the entrance increased, the contribution from animal surfaces (AGP; skin) decreased and the contribution from soil (EMP; non-saline) sources increased (Fig. 4b). Non-saline surfaces (EMP) also contributed as a source environment but remained stable except for a spike at a radius of 800 pixels.

A detailed description and interpretation of all facilities compared are beyond the scope of this report, but we provide a brief comparison of the present time-series SAF data with previous 100-swab KatharoSeq JPL-SAF data, one neonatal intensive care unit, an abalone-rearing facility, and three International Space Station (ISS) environmental data in characterizing a range of low-biomass environments and relating them to one another (Fig. 4c, d) [1]. Within each of the five environments, the extremely clean abalone-rearing facility exhibited entirely different microbial profile with the rest of the facility floors. Even though all other facilities look similar with reference to microbial diversity (Fig. 4c), detailed characterization of the weighted Unifrac analysis showed differential microbial diversity composition on neonatal intensive care unit and ISS surfaces when compared to JPL-SAF time-series samples. When compared with previous JPL KatharoSeq study sampled 100 swabs on one day of the same facility, this present JPL time-series data points differed in their microbial community composition (Fig. 4d).

**Fig. 5:**
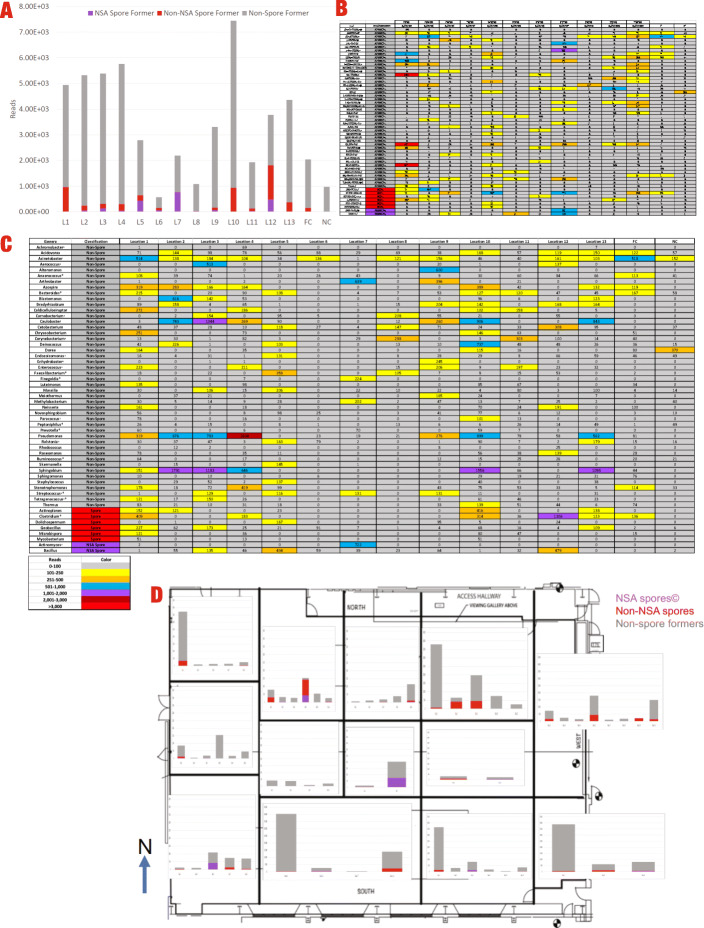
**A** Total PMA reads per location of genera with greater than 100 total reads across all samples. Genera are described as non-spore(gray), non-NSA spore(red), and NSA(purple). Field Control (FC) and Negative Control (NC) composition are also displayed. **B** Temporal heatmap of PMA-treated reads of genera with greater than 100 total reads across all samples. Genera are described as non-spore(gray), non-NSA spore(red), and NSA(purple). (◦ = facultative, * = anaerobic) **C** Spatial heatmap of PMA-treated reads of genera with greater than 100 total reads across all samples. Genera are described as non-spore(gray), non-NSA spore(red), and NSA(purple). **D** Spatial PMA reads by sampling location. Individual bars represent a single sample collected at a given location. Genera are described as non-spore (gray), non-NSA spore (red), and NSA (purple)

In the PMA-treated samples with greater than 100 total reads across all samples, a total of 46 non-spore forming genera and 8 spore forming genera were identified (Fig. 5). Across the non-control PMA-treated samples, the reads broke down into 4.28% (NSA spore-formers), 9.66% (non-NSA Spore-formers), and 86.05% (non-Spore-formers). The ten most predominant genera identified in PMA-treated samples were *Sphingobium, Pseudomonas, Caulobacter, Clostridium, Acinetobacter, Azospira, Bacillus, Deinococcus, Acidovorax, and Arthrobacter.* Of the ten most prominent overall genera, only two (*Clostridium* and *Bacillus*) were spore-formers. The eight spore forming genera were *Clostridium, Bacillus, Actinoplanes, Geobacillus, Actinomyces, Dolichospermum, Microbispora, and Mycobacterium.*

The *Bacillus* and *Actinomyces* genera were the only two NSA spore-formers detected in the PMA samples. *Bacillus* was the most temporally frequent NSA spore-former, which had more reads than *Actinomyces* in sampling sessions 2, 3, 4, 5, 6, 7, 10, and 11. Alternatively, a total of six non-NSA spore-formers (*Actinoplanes, Clostridium, Dolichospermum, Geobacillus, Microbispora, Mycobacterium*) were detected in the PMA samples. *Geobacillus* was the most temporally abundant non-NSA spore-former with the most non-NSA spore-former reads in the 1st sampling sessions. A total of 46 non-spore-formers were detected in the PMA samples. The most abundant non-spore genera varied for each sampling session; however, *Acinetobacter* did have the most reads in 2nd and 4th sampling sessions.

Comparison of NSA spores, non-NSA spore-formers, and non-spore-formers did not exhibit any consistent microbial population pattern (Fig. 5a). However, sampling sessions 4, 6, and 8 had higher reads of NSA spores compared to non-NSA-spore-formers. In every other sampling session, non-NSA-spore reads were greater than NSA spore reads. Additionally, reads of non-spore-former were greater than the combined reads of NSA spore and non-NSA-spore categories for every sampling session. The heatmap of the amplicon sequencing reads computed with the reads of NSA spores and non-NSA spores in Fig. 5b, c clearly shows that reads associated with spores were less than 100 reads in controls (except in 1 field control) and were most abundant when samples collected during 8th sampling sessions. Spatial and temporal distributions of the viable microbial population (PMA-treated samples) over 13 locations are depicted in Fig. 5d. Locations #5, 7, and 8 showed a higher presence of spores detectable by the NSA method than non-NSA spores. A higher incidence of non-NSA spore-formers was noticed in other locations, confirming that the NSA method misses the majority of these spore-formers which might need other optimal cultivation conditions for their growth. In addition, it cannot be ruled out that these spore-forming microorganisms might have been in the vegetative cell state and were killed during the 80°C; 15 min heat-shock procedure of the NSA method. It is also noteworthy to mention that all of these locations had more non-spore-forming members when compared to spore-formers.

*Bacillus* was the most spatially abundant NSA spore-former, with its highest total NSA spore-former reads in Location 12. *Geobacillus* was the most spatially frequent non-NSA spore-former, with the most non-NSA spore-former reads from Location 1. The most spatially abundant non-spore-former was *Sphingobium,* with high reads in Location 10 (Fig. 5c).

No NSA spore-formers were detected in the PMA field controls. Only *Bacillus* was detected in the PMA negative controls (2 reads). The most abundant non-NSA spore-formers in controls was *Clostridium* in PMA field controls (136 reads) and *Geobacillus* in PMA negative controls (6 reads). The most abundant non-spore-formers were *Acinetobacter* in PMA field controls (518 reads) and *Dorea* in PMA negative controls (370 reads) (Fig. 5b, c).

#### Single-cell genome sequencing

**Fig. 6:**
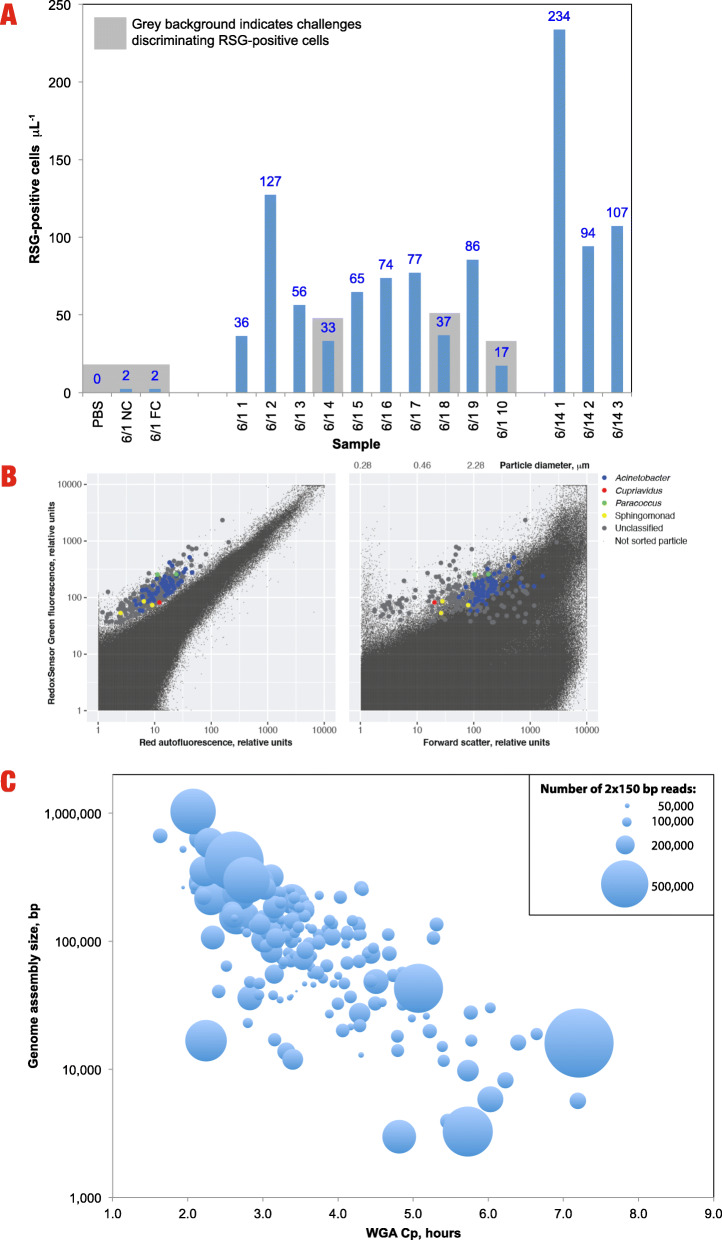
**A** RSG-positive cells per uL identified in various samples processed with fluorescent-activated cell sorting. **B** Flow cytometric characteristics and taxonomic affiliations of individual, RedoxSensor Green-positive cells from sample 2016-07-12-1A. **C** Genome assembly size by whole genome amplification Cp (hours)

As described previously in Hendrickson et al. (2017), 25 samples were analyzed via FACS to estimate the number of viable microorganisms. Viable cells, as identified by FACS, were randomly sorted into 384-well microplates, with 317 individual cells collected from each sample tested (Fig. 6a). For this study, sample 2016-07-12 Location #1 was chosen for proof of principal of the single-cell genomics analysis and was the only sample that was analyzed with all three methods (NSA, amplicon sequencing, and single-cell genome sequencing). This sample was chosen because it was located closer to the entrance of the cleanroom and also documented to have higher viable bioburden (8.8 x 10^5^ viable cells per m^2^) to analyze. Furthermore, the average spore counts on this sample was 35 spores per m^2^ of the surface area, giving the highest spore to viable microorganisms ratio of 12,091 [23]. A combination of genomic sequencing and PCR screens of the 16S rRNA gene of one of the microplates identified taxonomic affiliations of 78 of the 317 generated SAGs (Fig. 6b). Among the identified SAGs, 72 were identified as *Acinetobacter*, 3 as *Sphingomonads*, 2 as *Paracoccus*, and 1 as *Cupriavidus*. Additional genera, identified by the 16S rRNA gene PCR screens of two additional SAG microplates, included *Microvirga,* and *Novosphingobium* were also found in the SCG method. The size, florescence, and identity of RedoxSensor Green-positive cells are shown in Fig. 6b.

## Discussion

From the Viking mission in 1975 until the most recent Perseverance mission in 2020, NASA has utilized spore counts as a proxy to estimate the viable microbial burden on spacecraft destined for landing on Mars. However, due to the nature of the assay, only those microbes that can survive heat shock and grow on TSA plates are accounted for. With advancements in next-generation sequencing and single-cell genomics, we are better equipped to detect/monitor the inherent microbiome of spacecraft assembly cleanrooms that can help inform policies and protocols to minimize bioburden populations able to survive interplanetary travel and contribute to potential forward contamination risk of spacecraft hardware. The analysis provided by these molecular methods can also help determine whether these new technologies should now be used by NASA as a better measure of cleanliness or whether the established NSA is a suitable proxy for estimating the potential microbial contamination of spacecraft components. In this study, Sanger sequencing of NSA isolates along with amplicon sequencing and single-cell genomics allowed us to perform a temporal and spatial analysis of the microbial community composition over the 6-month period of collected samples. Additional shogun metagenomic analysis to infer functional pathways was not feasible given the low biomass samples from SAF (<1 pg per m^2^) which were far less than the amount required (~100 pg) for this additional analysis.

A previous study demonstrated that NSA and a DNA microarray assays did not show a statistically significant correlation between the cultivable spore counts obtained from a sample and the degree of bacterial diversity present [6]. DNA microarrays require a priori information of the microorganism and thus underestimate the correlation of spores and microbial diversity present in a given sample. It was also reported by these authors that the NSA spore-formers were not detected in the DNA microarray analysis due to the poor DNA extraction during that time.

While the NSA is aimed to be a proxy method to identify biological cleanliness on spacecraft surfaces, it can detect only a select and limited representation of the microbial population, specifically spore-formers cultivable under certain conditions [9, 17, 20, 23]. This was reflected in our results as ~97% of isolates were identified as spore forming bacteria. The other 3% of isolates were non-spore-formers but appeared to withstand 80°C for 15 min. The spore population was 3.6 x 10^1^ CFU per m^2^ of the SAF surface area which constituted ~20% of the total cultivable population [23]. A previous study that utilized ATP, PMA-qPCR, and FACS-based viable microbial burden analyses estimated the NSA was 10^2^–10^4^ fold less than the total bioburden present [23].

The NSA’s ability to identify a limited microbial population was apparent when compared with the 16S rRNA gene amplicon sequencing analysis that identified 1250 genera after PMA treatment (Fig. 3c–e). Although the 16S rRNA gene amplicon sequencing analysis detected 1250 viable genera, only 54 genera were detected with >100 total reads. Of the top 10 genera with the most reads, only two were reported to form spores. This contrasted with the 97% spore forming isolates identified in with NSA approach and confirmed that spore-forming microorganisms constituted a fraction of total bacterial population. Several spore-forming bacterial species that were dominant (e.g., *Geobacillus*, a thermophilic species) were not detected by NSA method because of its mesophilic cultivation conditions. Additionally, the OTUs with >100 reads included 8 spore forming genera whereas 12 spore forming genera were isolated via NSA. This might be due to the phylogenetic analysis resolution where 1.5 kb length of 16S rRNA gene fragments was used for identifying NSA isolates versus ~150-bp length of V4 variable region for amplicon sequence-based OTU calling. The V4 variable region of all 12 species isolated via NSA (*Bacillus, Brevibacillus, Gracilibacillus, Oceanobacillus, Paenibacillus, Rummeliibacillus,* and *Virgibacillus*) was conserved, hence it was not possible to resolve the inter-genus speciation and amplicon sequencing that might have identified them as *Bacillus.* However, sequences of the members belong to other spore-forming genera including *Bacillus* (aerobes), *Clostridium* (anaerobes)*, Actinoplanes* (oligotrophs)*, Geobacillus* (thermophiles)*, Actinomyces* (anaerobes)*, Dolichospermum* (cyanobacteria)*, Microbispora* (endophyte)*,* and *Mycobacterium* (slow-grower) were retrieved via the amplicon sequencing method. Identification of these non-NSA spore-forming genera was possible due to the high variation in V4 variable region. Hence, if the diversity of spore-forming microorganisms is the main goal, when amplicon sequencing is employed, the use of a universal house keep gene like *gyr*B [67] or functional genes like spore photoproduct B subunit [68] or sporulation genes [69] should be examined. The universal *gyr*B primer has been successfully used to differentiate the members of the genus *Bacillus cereus—anthracis* clade where full length 16S rRNA gene (~1.5 kb) showed 100% sequence similarities [67].

The temporal and spatial analysis of the amplicon sequences analysis showed variations in relative abundance of the genera that are NSA spore-formers (aerobic mesophilic members), non-NSA spore-formers (all spore-forming members exhibiting growth irrespective of cultivable conditions), and non-spore forming bacteria. Overall, the variations in reads showed that the non-spore-formers made up a significant percentage (~86%) of the reads in PMA-treated samples, while the NSA spore-formers and non-NSA spore-formers made up much smaller percentages or ~4% and ~10%, respectively. Temporally, these percentages varied from samples collected at various sampling session intervals (~15 days), represented by a large range for non-spore-formers (64–99%), NSA spore-formers (0 to 35%), and non-NSA spore-formers (1 to 22%). The percentages varied widely from one sampling session to another; however, a similar percentage breakdown of populations was observed between the 10th and 11th sampling session: non-spore-former (89 ➔90%), NSA spore-former (1➔2%), and non-NSA spore-former (10➔8%). The largest percentage of each population was observed on different time periods with non-spore-formers on 9th sampling (99%), NSA spore-formers on 6th sampling (35%), and non-NSA spore-formers on 2nd sampling time (22%). Similar variations were observed spatially across the samples. Non-spore-formers made up the majority of reads at locations ranging from 52 to 96% with the largest percent of the population in location 2. NSA spore-formers had a range of 0 to 35% with the largest percentage found in location 7. The non-NSA spore-formers had a range of 0 to 35% with the largest percentage found in location 12. In fact, location 12 had the highest overall spore percentage (NSA + non-NSA spore-formers) at 48%. Such a dynamic temporal and spatial distribution of all kinds of microorganisms might be due to the human traffic during this study.

When looking at raw reads, the three most abundant reads were detected on 1st sampling time on March 2, 2016, with three non-spore forming genera: *Caulobacter* (3805 reads)*, Pseudomonas* (5121 reads)*,* and *Sphingobium* (6111 reads). The most reads for non-NSA spore-former belong to *Clostridium* (995 reads) on 8th sampling period (June 28, 2016) and the most reads for a NSA spore-former were *Actinomyces* (722 reads) on this sampling session as well. Even though the cleanroom temperature was controlled, the higher 16S rRNA copy numbers from June 28 to Aug 16 2016 as measured by qPCR [23] might be potentially correlated to the outside temperature which was warmer in June to August 2016 (~90°F) when compared to March 2016 (~70°F) and is not statistically significant (data not shown).

Among the JPL-SAF cleanroom, locations 4, 7, 10, 11, and 12 had high human activities due to the assembly processes pertaining to the Mars 2020 mission subsystems. Since all personnel enter through location 1, despite thorough cleaning by the professional janitorial service, replenishment of microbial populations was predicted when compared to other locations and might be attributed to the microbial shedding by human. FACS exhibited higher counts (1 x 10^6^ per m^2^) of viable cells but PMA-qPCR copy numbers (5 x 10^4^ per m^2^) were not much higher in location #1 when compared to other locations [23]. The higher FACS counts compared to qPCR copy numbers might be due to potential loss during DNA extraction before amplicon sequencing whereas cells were counted in FACS method. *Pseudomonas* had the most non-spore forming reads with 2604 reads at location 4. The non-NSA spore-former reads of *Clostridium* (1306) were high at location 12, and the NSA spore-former *Actinomyces* (722) reads were highest at location 7. Since locations 5, 9, and 13 were far from human activities, fewer microbial reads were observed. The cleaning procedure was the same for all the locations where janitorial crew maintain the cleanliness of the JPL-SAF as per established protocol; however, due to the presence of hardware in locations 4, 7, 10, and 12, access to these locations might have been limited for cleaning.

Additionally, we observed variations in the spore to non-spore ratio based on proximity to the entrance of SAF (Fig. 4a). At the entrance in SAF location 1, this ratio was ~13 spore-formers to 1 non-spore-former. This ratio decreased on the samples collected farther from the entrance. Only one sampling site (location 4) showed an approximate equal ratio between spore-formers and non-spore-formers. On average, all other locations had a higher percentage of spore-formers compared to non-spore-formers. This change to spore to non-spore ratio could potentially indicate more hardy conditions near the entrance due to cleaning frequency. A previous study in the SAF compared microbial communities of 100 locations within the facility at a single time point [1]. When comparing this study to the previous SAF samples and additional built environments, it was clear that PMA treatment (and thus live vs. dead cells) in the SAF facility was a major discriminatory driver to community composition (Fig. 4c). Samples from the SAF facility clustered more closely to samples taken from the ISS compared to samples from earthly settings such as a hospital and building environments (Fig. 4c). Other built environments like JPL-SAF and the ISS, which have much more controlled access compared to unlimited access gained by the patients in hospitals and workers in office buildings might explain why JPL-SAF closely resembles the ISS with reference to microbial diversity profile. Even then, when the Unifrac analysis (Fig. 4d) was computed, a clear difference among ISS and JPL-SAF time-series data points was noticed.

Although the scope of this project only allowed for one sample to be analyzed using single-cell genomics, it did show differences from the other two analysis methodologies. The analyzed sample, 2016-07-12, location 1, identified 5 genera, all of which are described as non-spore forming microorganisms. *Acinetobacter, Sphingobium,* and *Paracoccus* species were found in both SCG and amplicon sequencing. However, members of the genera *Pseudomonas, Caulobacter,* and *Azospira* were found only in the amplicon sequencing approach whereas members of *Cupriavidus, Microvirga,* and *Novosphingobium* were found only in the SCG method. The FACS-SCG approach was able to quantify, determine cell sizes and recover genomic sequences of viable microbial cells from most of the analyzed SAF samples, without the need for cultivation.

**Fig. 7:**
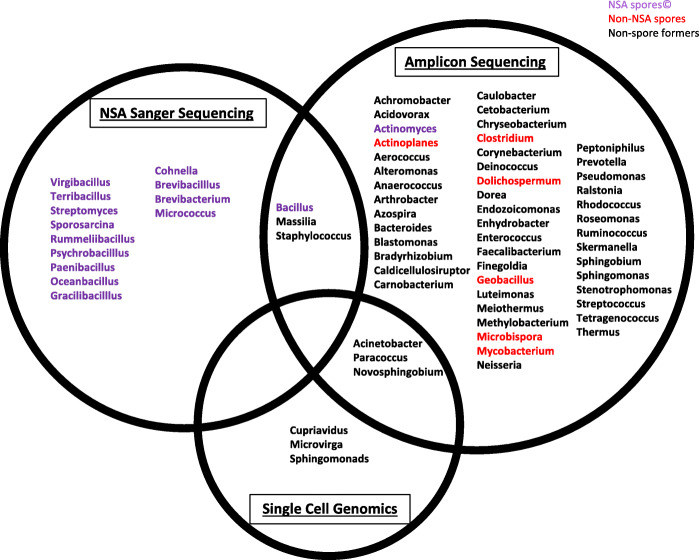
Venn diagram of identified genera in the three different methodologies used; Sanger sequencing, amplicon sequencing, and single-cell genomics. Purple text indicates NSA spore-formers and red text indicates all non-NSA spore formers

In this study, we observed 6 genera that were identified in two different methodologies and 64 genera only identified with one methodology (Fig. 7), many of which have been previously found in spacecraft cleanrooms [2, 7, 9, 17, 19, 20, 70–73]. Only 3 genera profiled via amplicon sequencing were also present in the SCG sequencing. This might be attributed to only one sample being analyzed by SCG or from a PCR bias where a low number of DNA fragments were only amplified in amplicon sequencing method. Although Fig. 7 shows the genera identified in each methodology, it is difficult to completely compare the Sanger sequencing of culture-based isolates with the above molecular techniques because of inherent selective process of the NSA and cultivation compared to the other molecular methods that used PMA-treated DNA.

## Conclusion

The SAF is a unique artificial environment with oligotrophic conditions that result in a unique microbial population. All three methodologies provide a unique perspective of the total microbial population, and this study has shown that *observed bioburden abundance and diversity are heavily affected by choice of analysis method*. To understand the overall microbial population, multiple methodologies should be considered to obtain a more complete picture of the microbial composition. The utilization of both the NSA and molecular methods is necessary to provide the scientific community with a more complete picture of the bioburden fractions that may survive interplanetary travel (desiccation, vacuum, low-nutrient, etc.) and proliferate in the extraterrestrial environmental conditions (anaerobes, psychrophiles, radiation resistant microorganisms, etc.) while also linking those findings to the long bioburden history documenting the bioburden that could be present on robotic missions.

## Supplementary Information

**Additional file 1: Figure S1.** Comparison of microbial community composition between SAF location. First two-principle components of the Compositional Tensor Factorization (CTF) biplot, dots colored by most prevalent phyla being Rhizobiales (Proteobacteria) (red) and Bacillales (Fimicutes) (blue), arrows colored by SAF location radial distance from entrance (**A**). Log-ratio of lowest common ancestor grouped taxonomic groupings in the biplot correlated to radial distance from the SAF entrance (**B**). **Figure S2.** Seven contaminants removed from analysis which were present in 100% of the controls and had a positive ratio compared to control:PMA- samples. **Table S1.** Effect size in ordinations from PMA treatment and the collection timepoint. **Table S2.** Sample size, number of groups, test statistic, and P-value of weighted UniFrac, Unweighted UniFrac, RPCA, and CTF.

## Data Availability

The data presented in this manuscript are available in the NCBI Sequence Read Archive under the accession no. PRJEB40954. The 16S rRNA sequences of the isolates were deposited in the NCBI GenBank under accession no.: MW130960 to MW131089. Associated 16S feature tables are publicly available in Qiita (qiita.ucsd.edu) [73] under study ID 10689 and analysis code can be found at https://github.com/knightlab-analyses/SAF-PP-JPL.
