# Supplementary material for: Clean room microbiome complexity impacts planetary protection bioburden
Source: Microbiome. 2021 Dec 4;9:238. doi: 10.1186/s40168-021-01159-x (PMC8643001; doi:10.1186/s40168-021-01159-x)
Supplement: Supplementary file 2 — Additional file 1: Figure S1. Comparison of microbial community composition between SAF location. First two-principle components of the Compositional Tensor Factorization (CTF) biplot, dots colored by most prevalent phyla being Rhizobiales (Proteobacteria) (red) and Bacillales (Fimicutes) (blue), arrows colored by SAF location radial distance from entrance (A). Log-ratio of lowest common ancestor grouped taxonomic groupings in the biplot correlated to radial distance from the SAF entrance (B). Figure S2. Seven contaminants removed from analysis which were present in 100% of the controls and had a positive ratio compared to control:PMA- samples. Table S1. Effect size in ordinations from PMA treatment and the collection timepoint. Table S2. Sample size, number of groups, test statistic, and P-value of weighted UniFrac, Unweighted UniFrac, RPCA, and CTF. [file 40168_2021_1159_MOESM2_ESM.pptx]

## Slide 1
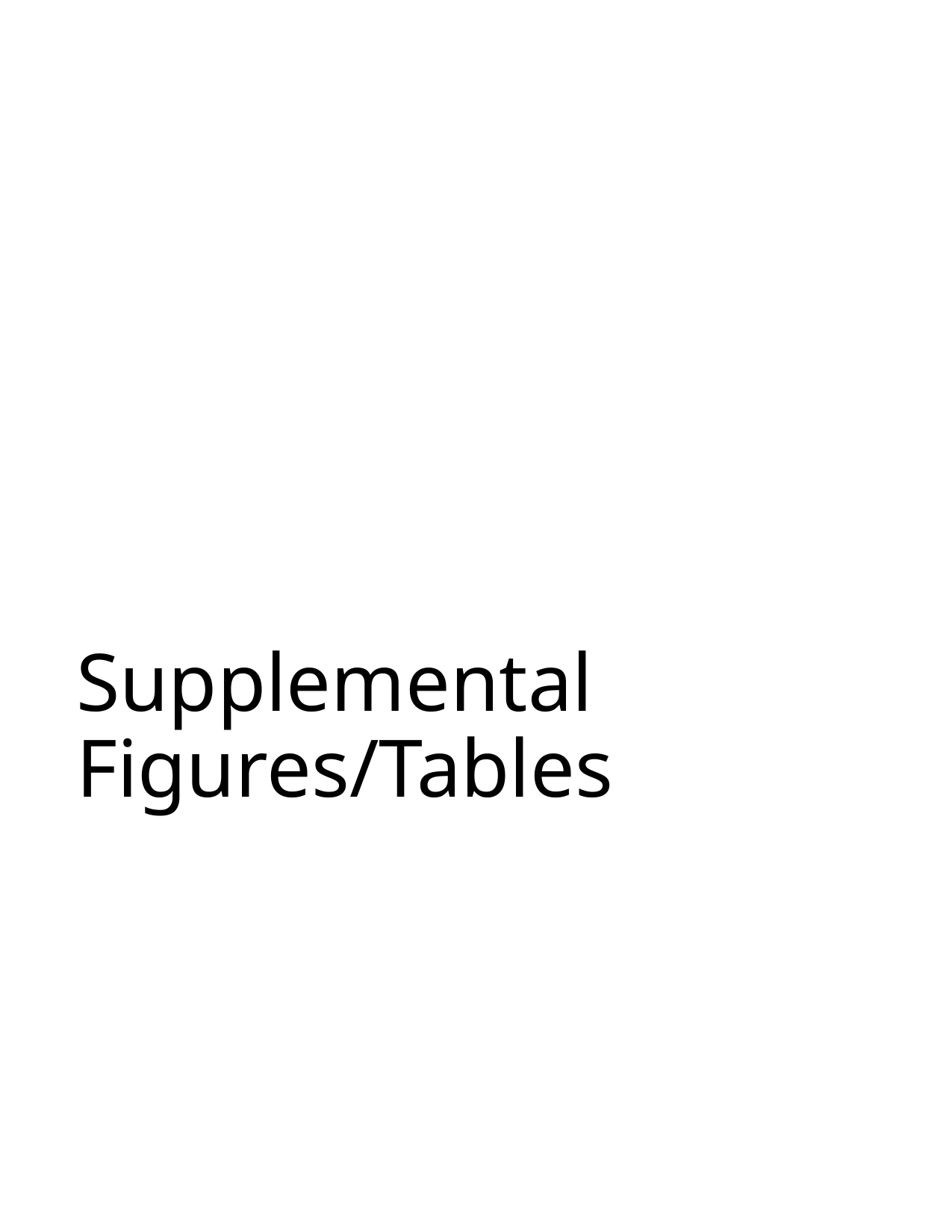

# Supplemental Figures/Tables

## Slide 2
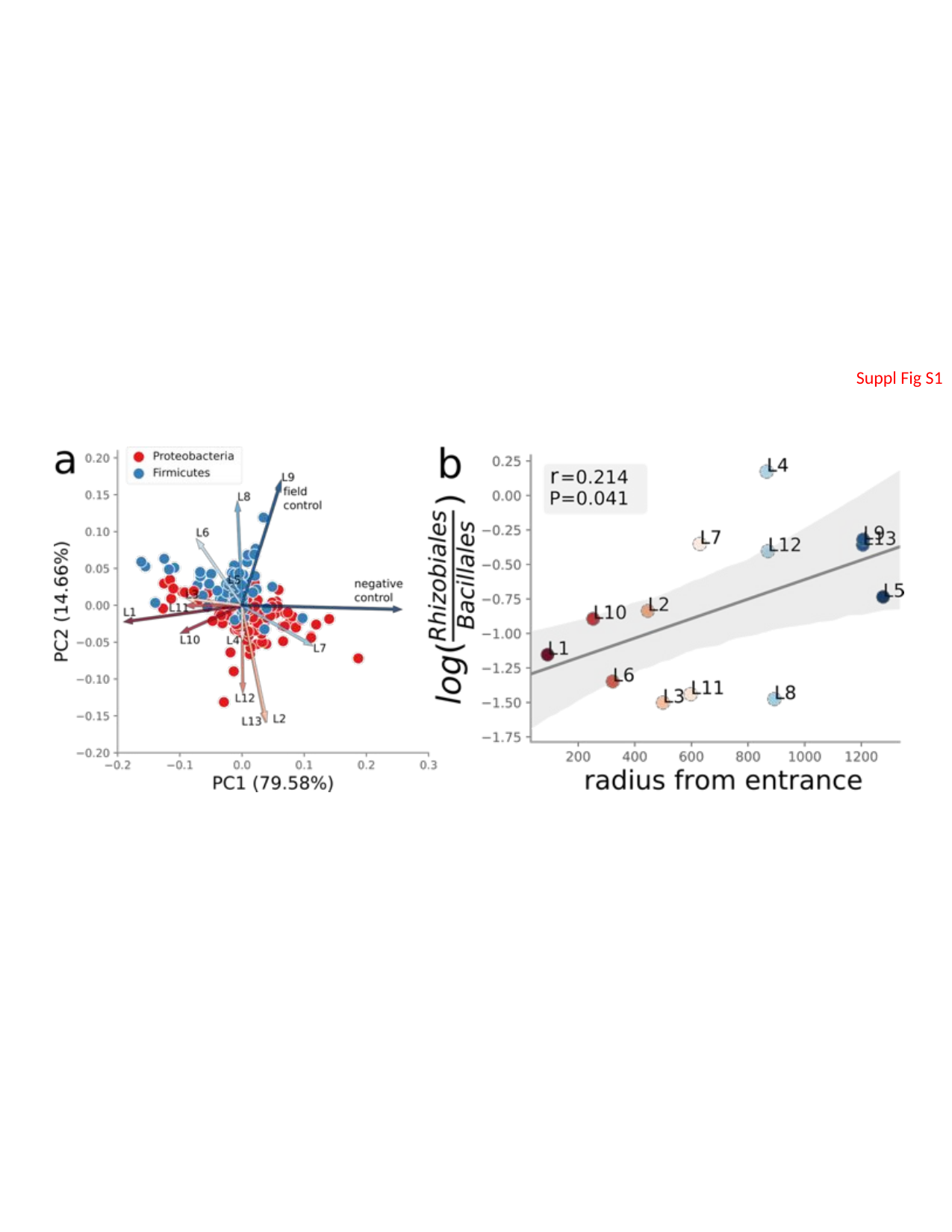

Suppl Fig S1

## Slide 3
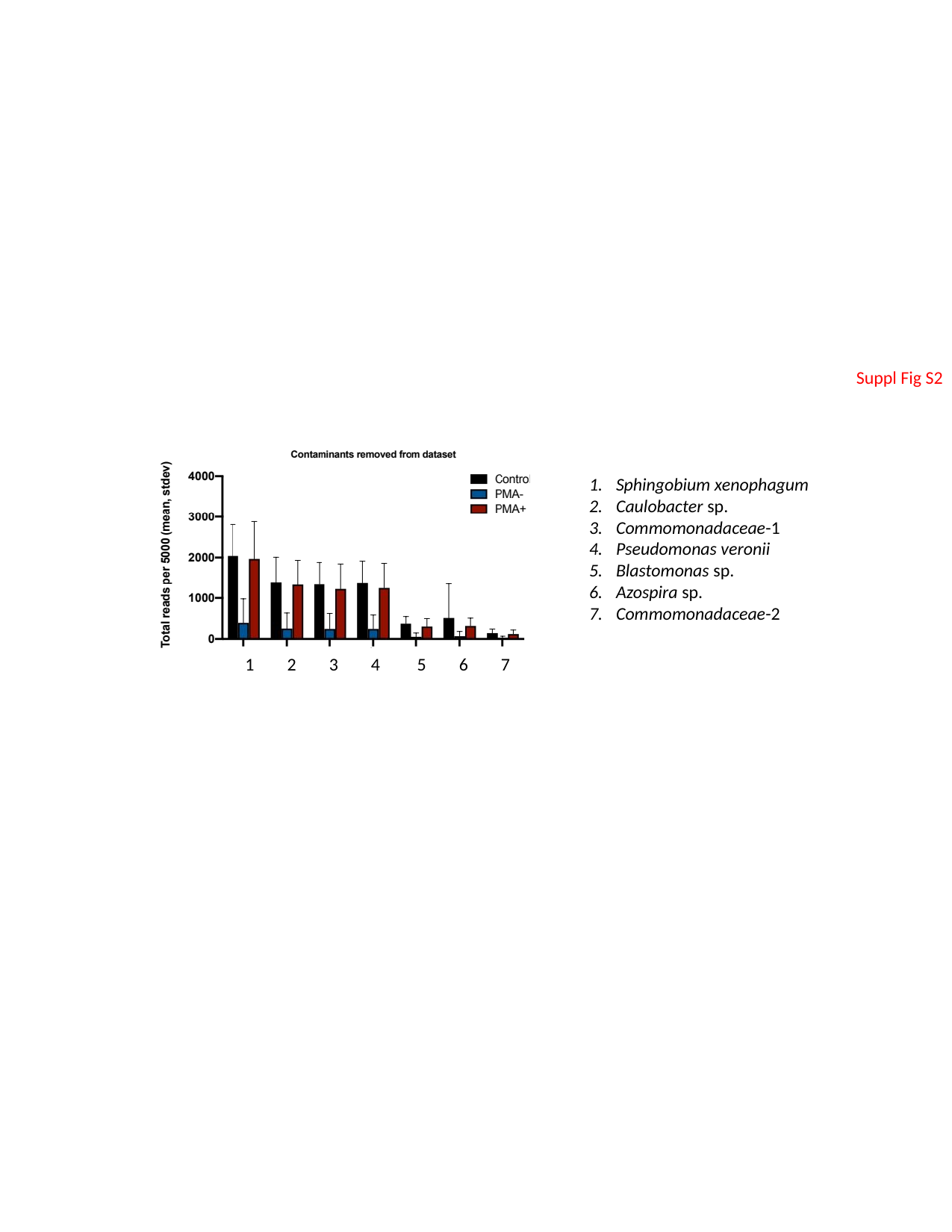

Suppl Fig S2
Sphingobium xenophagum
Caulobacter sp.
Commomonadaceae-1
Pseudomonas veronii
Blastomonas sp.
Azospira sp.
Commomonadaceae-2
1 2 3 4 5 6 7

## Slide 4
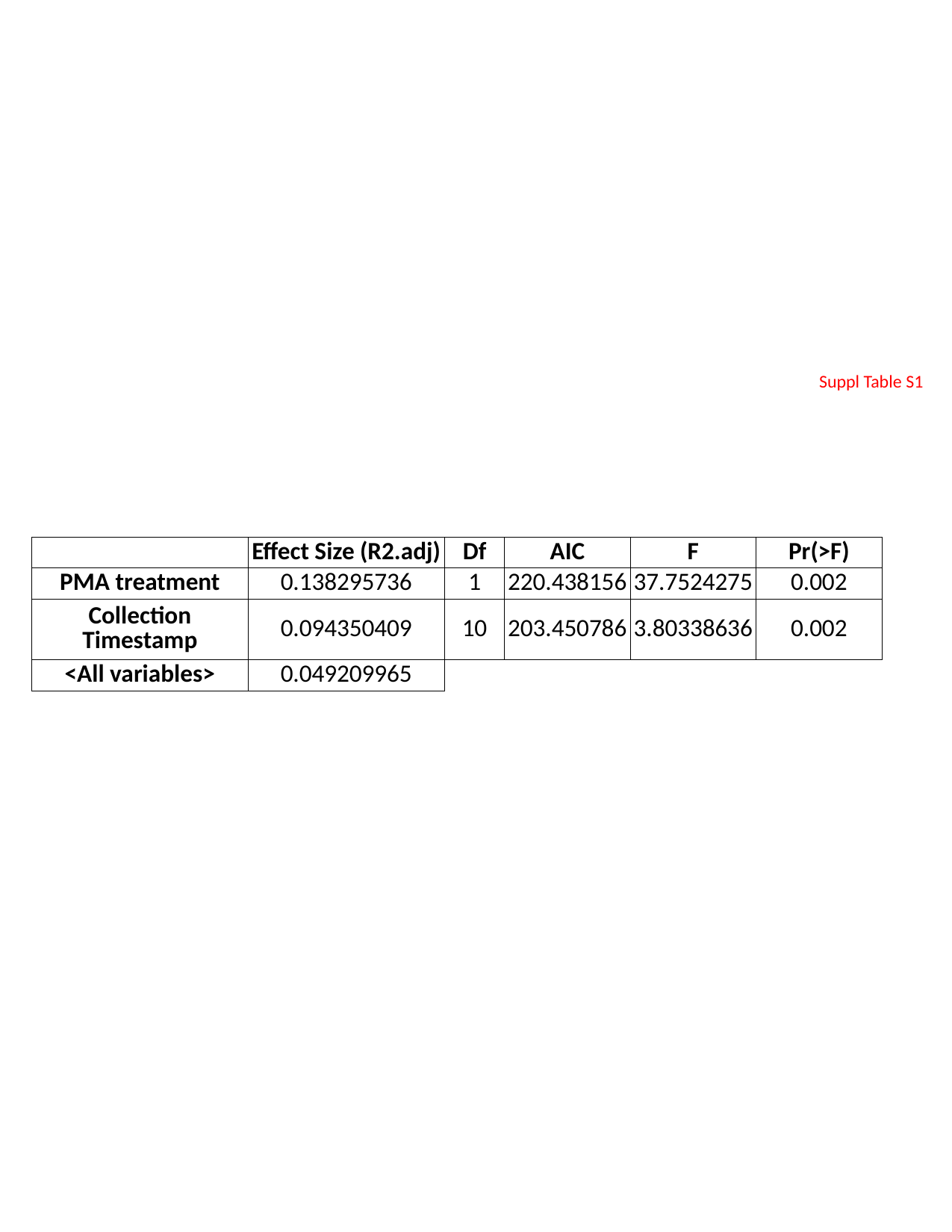

Suppl Table S1
| | Effect Size (R2.adj) | Df | AIC | F | Pr(>F) |
| --- | --- | --- | --- | --- | --- |
| PMA treatment | 0.138295736 | 1 | 220.438156 | 37.7524275 | 0.002 |
| Collection Timestamp | 0.094350409 | 10 | 203.450786 | 3.80338636 | 0.002 |
| <All variables> | 0.049209965 | | | | |

## Slide 5
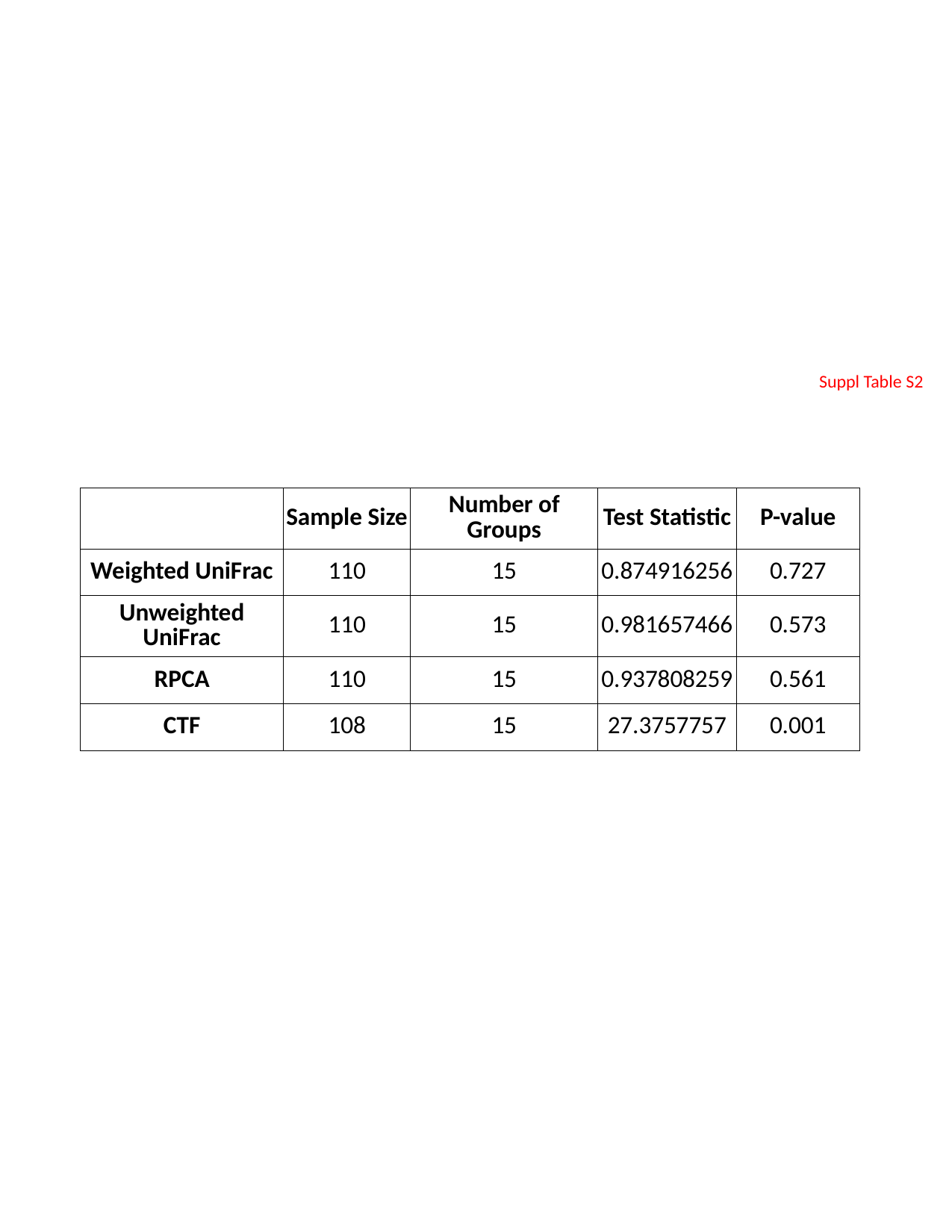

Suppl Table S2
| | Sample Size | Number of Groups | Test Statistic | P-value |
| --- | --- | --- | --- | --- |
| Weighted UniFrac | 110 | 15 | 0.874916256 | 0.727 |
| Unweighted UniFrac | 110 | 15 | 0.981657466 | 0.573 |
| RPCA | 110 | 15 | 0.937808259 | 0.561 |
| CTF | 108 | 15 | 27.3757757 | 0.001 |
